# The Backbone of the College Finances

**Published:** 1920-06-26

**Authors:** 


### THE MATRONS' AND SISTERS' DEPARTMENT.

### THE BACKBONE OF THE COLLEGE FINANCES.

The keynote of the very happy meeting of the College which we chronicle this week was finance, and this was only fit, for the state of the treasury is the pulse of all voluntary associations. When it is strong the members are strong too, for financial support can only be attracted to causes which display vitality.

When the annual report was issued it showed an accumulated fund amounting to nearly £57,000, a considerable addition to last year's figures. Sir Arthur Stanley was, however, able to announce to the meeting that since the end of the financial year a further addition of something like £30,000 had been made in promises and in money. The College, therefore, is within about £13,000 of attaining the fund of £100,000 which, it has always been estimated, was the minimum which would enable it to carry out its great purposes with any security of permanence. This in itself is a great landmark.

Again, the Nation's Fund for Nurses set itself as its first great task the duty of raising £100,000, the interest of which—from £5,000 to £6,000—should be available for relieving nurses in distress, and especially such as had suffered in consequence of the war. Although the full figures have not yet come to hand of the Nation's Fund for Nurses, the Chairman was able to announce that a sum has been reached which is not very far short of the total required. The Nation's Tribute to Nurses is now an accomplished fact, and has become a permanent addition to the great benevolent resources of the country. Alas! the demands made upon it have far exceeded expectations, and it is clear that a capital sum considerably larger than that at first suggested is desirable, and, indeed, we might almost say indispensable.

In the raising of these sums nurses have shown themselves to be the backbone of the movement. Lady Cowdray and Miss May Beeman, to whom nurses owe so much, could never have done so well had not nurses themselves been the keenest to help in raising funds for the relief of their suffering sisters. The generous exertions of these two ladies, of Mrs. Martin Harvey, of Lord Burnham, and many others have crowned the movement with success, but it is essentially a nurses' movement, made by nurses for nurses. To have achieved their aim so swiftly in these hard times testifies to the great hold which nurses have on the generous public, but it testifies still more to their own zeal for their profession.

Nurses have contributed directly to the various funds initiated by the College no less than £51,000 since its foundation, and the Chairman is justly proud of this token of loyalty. It is now a moot point whether new members of the College should not be asked to pay a yearly contribution towards defraying the rapidly-growing expenses of their own organisation. Every year brings round fresh things which need to be carried through in the interests of members, and last year, owing mainly to the issue of the register, the expenditure exceeded the ordinary income by nearly £800. Sir Arthur Stanley, with the caution born of long experience, forecasted that every year there would be exceptional claims on the College to the tune of some £1,000. So long as it remains in the van of nursing progress this is a very safe forecast, and it certainly suggests that members may very easily supply the funds for such extraordinary exertions by contributing a small yearly subscription. By the articles of association original members cannot now be asked for a subscription; but a large proportion of them would certainly pay voluntarily, and new members who join what is now an assured success, and obtain benefits far in excess of what could even be contemplated at the outset, would consider it entirely reasonable to pay a small sum annually, or compound by payment of a life subscription. The policy of letting one guinea cover all liabilities proved well adapted for the purpose of starting an entirely new organisation. It does not follow that it is adapted to the needs of the College as it stands after years of incessant work on the part of its founders, and we trust steps may be taken forthwith so to alter the articles of association that newcomers may do their share in consolidating the work so finely initiated for their benefit.

Sir Arthur Stanley, who had several notable announcements to make, was never more warmly applauded than when he revealed the approaching possession by the College of its own hall and central residential club. There can be no doubt that handsome, spacious, and centrally situated premises are the finest object-lesson which can be afforded to the members of a successful organisation. It is high time that the College entered into the enjoyment of this well-earned satisfaction

